# A population-based model for priority setting across the care continuum and across modalities

**DOI:** 10.1186/1478-7547-4-6

**Published:** 2006-03-28

**Authors:** Leonie Segal, Duncan Mortimer

**Affiliations:** 1 Centre for Health Economics, Monash University, Melbourne, Australia

## Abstract

**Background:**

The Health-sector Wide (HsW) priority setting model is designed to shift the focus of priority setting away from 'program budgets' – that are typically defined by modality or disease-stage – and towards well-defined target populations with a particular disease/health problem.

**Methods:**

The key features of the HsW model are i) a disease/health problem framework, ii) a sequential approach to covering the entire health sector, iii) comprehensiveness of scope in identifying intervention options and iv) the use of objective evidence. The HsW model redefines the unit of analysis over which priorities are set to include all mutually exclusive and complementary interventions for the prevention and treatment of each disease/health problem under consideration. The HsW model is therefore incompatible with the fragmented approach to priority setting across multiple program budgets that currently characterises allocation in many health systems. The HsW model employs standard cost-utility analyses and decision-rules with the aim of maximising QALYs contingent upon the global budget constraint for the set of diseases/health problems under consideration. It is recognised that the objective function may include non-health arguments that would imply a departure from simple QALY maximisation and that political constraints frequently limit degrees of freedom. In addressing these broader considerations, the HsW model can be modified to maximise *value-weighted *QALYs contingent upon the global budget constraint and any political constraints bearing upon allocation decisions.

**Results:**

The HsW model has been applied in several contexts, recently to osteoarthritis, that has demonstrated both its practical application and its capacity to derive clear evidenced-based policy recommendations.

**Conclusion:**

Comparisons with other approaches to priority setting, such as Programme Budgeting and Marginal Analysis (PBMA) and modality-based cost-effectiveness comparisons, as typified by Australia's Pharmaceutical Benefits Advisory Committee process for the listing of pharmaceuticals for government funding, demonstrate the value added by the HsW model notably in its greater likelihood of contributing to allocative efficiency.

## Background

The primary task of priority setting is to determine desirable resource shifts – health services to be expanded and those to be contracted – to support the achievement of health and other social objectives. A number of priority setting models have been employed by third-party fundholders to this end including Program Budgeting and Marginal Analysis (PBMA) and various approximations to the QALY league table approach. A number of recent reviews describing key features of existing priority setting models and their application are available for those unfamiliar with the literature [1-5].

While these models have the capacity to identify desirable resource shifts, the challenges of funding and delivery under a global budget have led to a fragmentation of the health sector into 'program budgets' or 'budgetary silos' – typically defined by modality or disease-stage. One consequence of this fragmentation into program budgets is that allocation and re-allocation of resources commonly occurs *within*, rather than *between*, budgetary silos. Where mutually exclusive interventions are funded under different program budgets, decision-makers may consider only options within their ambit, failing to identify more cost-effective options outside their program area. Competing interventions funded under different budgets may also be required to clear different hurdles, such that funding may be easier to obtain for cure than for prevention, for drug-therapy than for counselling, or for inpatient care than for treatment in a community setting. Unless any such differences in the probability of funding can be linked to some argument in the objective function that systematically varies by program budget, then efficiency gains are available by shifting resources between budgetary silos. Further, complementarities between modalities in the treatment of a particular disease/health problem may be ignored, such that composite interventions or coordinated approaches to prevention or treatment must rely on ad hoc funding unless their component parts can be shown to be independently cost-effective.

In short, an emphasis on priority setting at the level of program budgets, defined by modality or disease-stage may entrench allocative inefficiency – particularly between budgetary silos.

In this paper we describe an economic model that explicitly considers resource allocation across modality (medical, surgical, pharmaceutical, media, allied health etc.) and disease-stage (primary prevention, early diagnosis, management of established disease and end stage care/palliation). It is argued that this focus on allocation and re-allocation of resources across the care continuum and across modalities for each disease/health problem under consideration has the capacity to deliver allocative efficiency gains when compared to priority setting models that are predominantly concerned with the narrower question of allocation within a program budget. It is argued that, because of the disease/ health problem focus and the involvement of a broad range of interests – the model is also less likely to promote capture by a single interest group.

## Methods

### The model

The Health-sector Wide (HsW) priority setting model is designed to shift the focus of priority setting away from 'program budgets' that are typically defined by modality or disease-stage and towards well-defined target populations with a particular disease/health problem. The key features of the model are a disease/health problem framework, a sequential approach to covering the entire health sector, comprehensiveness of scope in identifying intervention options and reliance on objective evidence, but with a limited role for clinical experts and consumers. Since publication of an early version in 1994 [6], the HsW model has been refined and applied in several disease-areas. The current paper describes the refined model, discusses issues arising from application of the model that bear upon implementation, and compares the model to other economic models of priority setting.

The model categorises the entire health sector into diseases and health problems. The point of adopting this categorisation, which undoubtedly is a simplification, is to provide a framework that allows the health sector wide priority setting task to be staged. In essence, fragmentation of the health sector by 'program budgets' that are typically defined by modality, disease-stage or agency, is replaced by classification by disease/health problem which in large part equates with particular sub-populations. This approach supports a staged analysis as that the likelihood of sub-optimising is reduced by a process that requires a wide range of competing interventions to be considered in a single priority setting exercise. The logic of a priority setting approach that seeks to identify how most efficiently to reduce burden on sub-populations of disease/health conditions/risk factors derives from the object of health care, which is the health of populations, not the provision of health care as an end in itself. In contrast, analysing interventions within a specific delivery context – such as modality or agency – in effect presumes there are no substitutes outside that context. If that is not the case, the priority setting process will inevitably result in sub-optimising. That is while it may support an efficient distribution of funding within the nominated context (eg pharmaceuticals), it will almost certainly be inefficient in funding that context relative to others.

After imposing the disease/health problem categorisation, the model hypothesises that the disease burden can potentially be addressed by intervening through: i) primary prevention targeted at persons at risk, ii) early diagnosis (and treatment) of persons with undiagnosed diseases, iii) management of persons with established disease and iv) palliation for persons in the terminal phase. This categorisation is purposefully comprehensive, but recognises that for certain diseases there may be no feasible intervention at one or more disease stage.

This continuum of care within a specific disease/health problem defines the unit of analysis over which priorities are set, with the aim of including all mutually exclusive and complementary interventions for the prevention and treatment of that disease/health problem. That is to say, priority setting exercises *initially *proceed within the relevant disease/health problem column in Table 1 below, but is to eventually encompass the entire health sector via sequential or parallel application across columns.

**Table 1:** HsW (Health-sector wide) model framework

Disease stage *Target population*	Disease or health problem					Total budget allocated to disease stage
		
	Musculoskeletal	CVD	Cancers	Endocrine	Etc ........	
		
	OA RA	CHD stroke	Lung, Breast, CRC	IDDM NIDDM		
Primary prevention *Persons at risk*						
Early diagnosis *Persons undiagnosed*						
Management *Persons with established disease*						
Palliative care *Persons in terminal phase*						
Total budget allocated to prevention and management						

Notes: OA osteoarthritis, RA Rheumatoid arthritis, CVD cardio-vascular disease, CHD coronary heart disease, CRC colorectal cancer, IDDM insulin dependent diabetes mellitus (Type 1), NIDDM non-insulin dependent diabetes mellitus (Type 2).

Once a disease or health problem area has been selected, the model involves 10 distinct tasks to be completed by decision-makers and analysts, some of which proceed together rather than sequentially:

1. *Obtain a thorough understanding of the disease/health problem *– in terms of disease aetiology, normal disease progression and feasible points of intervention to reduce disease burden.

2. *Set up an Expert Panel *of clinicians and consumers to assist with access to, and interpretation of the clinical and health economics literature and for later dissemination of findings.

3. *Identify all intervention options at each disease stage *– proceeding cell by cell, down a column. Comprehensiveness in terms of modality, health delivery setting, target population, philosophy of care, occupation group/professional mix and method of delivery is paramount. The aim is to include all mutually exclusive and complementary interventions for the prevention and treatment of the disease/health problem under consideration. Interventions identified by horizon scanning should be included, as well as existing technologies, without regard, in the first instance to the quality of available evidence.

4. *Select interventions to include in the priority setting exercise *– From all possible interventions identified, select interventions for estimation and comparison of performance. The selection should aim for comprehensiveness, contingent upon constraints related to the availability of evidence. Any intervention that is currently funded or is likely to receive funding should be included. Where lack of evidence precludes formal evaluation, the identified data gaps should feed into research priorities – see also discussion under 7.

5. *Specify the measure of benefit *– The selected measure of benefit should allow comparison within and between columns in Table 1 above and reflect community values. Consider whether defined benefit should extend beyond the individual (to include impact on family members/the wider community) and whether health maximisation should be the sole concern. The QALY or value-weighted QALY – incorporating mortality, quality of life and other dimensions of benefit (such as equity) – are perhaps the most suitable composite outcome measures that support comparisons across disparate interventions.

6. *Collect evidence on costs and outcomes *primarily from the published clinical trial literature. Identify all published studies that provide evidence on outcomes and costs (resource use) for the interventions to be included in performance measurement. Locate and use studies of 'best' quality, with respect to study design, sample size, length of follow-up and reporting of pertinent interpretable outcomes, and the precision with which intervention and comparator are specified.

7. *Identify critical data gaps *in the primary clinical trial evidence and to estimate downstream and broader 'external' impacts. Use this information to inform research priorities. In the short-term, scenario analysis, informed by expert opinion may be used to estimate performance, recognising however, that where evidence is poor, estimated performance may be too imprecise to develop recommendations concerning desirable resource shifts.

8. *Calculate the cost-effectiveness of each intervention at each disease stage *– that is within each cell of Table 1, working down the relevant disease/health problem column. Use standard techniques of cost-effectiveness analysis [7,8] with an emphasis on achieving comparability in performance measurement. This inevitably means intermediate outcomes must be translated into final outcomes and modelling beyond published trial results.

9. *Compare the performance of interventions within and across disease stages *– within cell comparisons can potentially proceed using intermediate outcomes but between cell comparisons require a 'final' composite outcome measure such as the QALY or value-weighted QALY.

10. *Develop conclusions about desirable resource shifts *– identify desirable resource shifts based on the relative performance of competing and complementary interventions and sort these resource shifts by the level of supporting evidence. Interventions excluded from the performance measurement task due to a lack of evidence but still of interest (for instance because they are currently funded), should be listed alongside interventions for which performance measurement was possible.

## Implementation/Results

A number of challenges arise when applying the simple HsW model described above. First, a lack of objective evidence on effectiveness for the full range of intervention options (existing and potential) across settings, sub-populations and the care continuum may leave a potentially large number of interventions excluded from explicit performance measurement. Secondly, converting clinical and other outcomes into a common unit of benefit to support comparison of interventions across disease stages and across diseases is problematic due to data gaps and unresolved methodological questions. Thirdly, decision-makers frequently prefer to leave political, ethical or distributional objectives implicit rather than support explicit decision-rules based on objective evidence.

The extent to which these challenges impede implementation of the HsW model has been explored in applications to osteoarthritis [9], Type 2 Diabetes [10,11], hypertension and harmful life style behaviours [12,13]. These applications have shown that the HsW model is not distinguished from other priority setting models by its ability to overcome these problems. Rather, it is the sort of questions that the HsW model emphasises that distinguishes it from priority setting models that are predominantly concerned with the narrower question of allocation within a program budget. The disease/health problem framework of the HsW model supports explicit consideration of allocation and reallocation of funding across all mutually exclusive and complementary interventions for the prevention and treatment of a disease/health problem under consideration. The published applications demonstrate the robustness of the framework in this task and in developing recommended resource shifts across disparate modalities and across disease stages, which, if followed, should contribute to social objectives and that application across the entire health sector is feasible. (Recommendations have covered for instance, exercise, patient education, surgery, complementary medicines, prescription medicines, public health campaigns, primary care advice etc.).

That said, the translation of recommendations to actual resource shifts is dependent on the incentives in the health system and in particular whether funding arrangements are compatible with the health sector wide context and disease/health problem framework. The most compatible funding framework is a global budget for the (sub) population, managed by a single fundholder. The alignment of budgetary silos with the disease/health problem framework might also be successful. A mismatch between the funding framework and the priority setting framework requires a mechanism for shifting resources between budgets. This could plausibly be achieved by permitting one fundholder to 'buy-out' the budget of another fundholder provided suitable accountability mechanisms are in place. More generally, the very funding models that give rise to the observed distortions in the health service mix discourages any redirection of resources to address them. Whilst the HsW model is not distinguished from other priority setting models by its ability to overcome this problem, it does identify the cost of retaining funding silos. Setting priorities at the level of the program budget will typically fail to identify this major source of inefficiency.

In relation to consideration of interventions for which the evidence is extremely poor, the solution in the medium to longer term has to lie in the gathering of additional evidence. The alternatives are to adopt the status quo – to continue to fund unproven interventions, which in effect means adding to but not deleting interventions from the formulary (requiring an ever expanding program budget), or rely on the black-box of committee or working-group decision-making, allowing decision-maker values or expert opinion to determine the final list of priorities. Neither alternative is likely to promote efficiency. In the short term, a lack of data should not be allowed to paralyze the decision-making process. The HsW model avoids decision paralysis by retaining all competing and complementary interventions of interest in the choice set but differentiating interventions according to the level of supporting evidence. The extent of reliance on opinion and the sway of political imperatives in modifying the set of priorities is therefore made quite explicit when implementing the HsW model. The value of the HsW model is to identify data gaps, highlight the need for objective evidence and avoid any confusion between opinion-base and evidence-based decision-making.

### Comparison with other priority setting models

The HsW model has elements in common with other economic-based approaches such as QALY League Tables [2,14], PBMA [1,15-17] and generalised cost-effectiveness analysis [18,19]. A full description and critique of all competing priority setting models is beyond the scope of the current paper but the key features of three alternative economic models, together with a brief consideration of their strengths and weakness relative to the HsW model, are outlined below. See also the critique by Coast and colleagues [2], the report by Segal and Chen [3] and the discussion paper by Hauck and colleagues [4].

#### Limited/constrained comparisons

Typically in health economic evaluation a small number of interventions are compared, often drawing on the results of a single clinical trial or a meta-analysis of similar trials. The process for the listing of pharmaceuticals on government formularies, such as the Australian Pharmaceutical Benefits Schedule (PBS) is a well-known example of a restricted approach to priority setting. In order for drugs to be listed on the PBS (a mechanism to subsidise pharmaceuticals) pharmaceutical companies must submit an economic evaluation to support listing. Evaluations are prepared according to a set of published guidelines [8] and submissions are subject to independent review. Other treatment modalities are ineligible for listing.

The process ensures consistency in methods and high standard of analysis. But the guidelines define the main comparator narrowly, as another drug, normally of the same pharmacological analogue – if such a drug is listed on the PBS, or otherwise of the same therapeutic class. Comparison with 'standard medical management' occurs only on the rare occasion when there is no suitable comparator listed on the PBS. This restriction on scope means that drug treatment is rarely compared with alternative modalities and never with modalities that do not represent usual care. In the context of an open ended pharmaceutical budget (where supply responds without limit to demand, as applies in Australia), this process supports the increasing use of pharmaceuticals relative to other modalities. This will be the result unless the performance threshold, in terms of incremental cost-effectiveness ratio, that supports listing is set to reflect decision making elsewhere in the health sector. But it is more likely that decisions on pharmaceuticals will be made independently, without regard to the incremental cost-effectiveness ratios of competing modalities that could be obtained through a broader health sector wide approach to priority setting. In the absence of that broader context, limited cost-effectiveness comparisons are at clear risk of entrenching resource silos within program boundaries and modalities.

#### QALY league tables

QALY league tables in contrast attempt to be comprehensive, with the aim of comparing all current and possible health interventions in a single priority setting exercise. The most commonly cited example of QALY League Tables in action is the Oregon Experiment of the early 1990s – an attempt to identify a set of health services to be funded under the US Medicaid program for the people of Oregon [14]. The research program and subsequent recommendations ran into difficulties, due to lack of confidence in the cost and QALY estimates, but also a fundamental mistrust of the allocation of resources on the basis of cost/QALY. The difficulty posed by the sheer scope of the exercise is substantial, especially if the analysis is to be truly at the margin and to consider all possible target populations and intervention options [21].

Of the four alternative economic models considered here, QALY league tables share the greatest commonality with the HsW model. It is therefore worth highlighting exactly how the HsW model addresses some of the difficulties experienced when using QALY league tables for priority setting. The HsW model addresses issues related to the lack of confidence in cost/QALY estimates by permitting the measure of benefit to capture ethical or distributional concerns, by a reliance on objective evidence with respect to all arguments in the objective function for interventions included in performance measurement tasks, and by making explicit the lack of evidence associated with interventions excluded from the performance measurement but included in the priority setting exercise. The HsW model addresses the vastness in scope and size of task, by dividing the priority setting exercise into a succession of manageable research activities, covering all mutually exclusive and complementary interventions for the prevention and treatment of a single disease/health problem. That is, by adopting the disease/health problem framework described above.

#### Generalised Cost-Effectiveness (G-CE) analysis

was developed to resolve issues with regards the transferability and policy applicability of cost-effectiveness analysis (CEA) that might limit or preclude its use and re-use for sector-wide priority setting [18,19]. More specifically, Murray and colleagues [19] provide a set of guidelines for G-CEA with two potential situations in mind: (i) "CEA of a wide-range of interventions ...to inform a specific decision-maker facing a known budget constraint, a set of options for using the budget and a series of other (ethical or political) constraints" (p237), and (ii) "CEA of a wide-range of interventions ...to provide general information on the relative costs and health benefits of different technologies that are meant to contribute through multiple channels to a more informed debate on resource allocation priorities" (p237). Given the alignment of budgetary silos with the disease/health problem framework or the existence of a global budget managed by a single fundholder for the (sub) population under study, the application of the HsW model would be equivalent to the first situation. However, in the presence of a mismatch between the funding framework and the priority setting framework, the HsW model would have more in common with the second situation – informing debate by identifying the cost of retaining budgetary silos and a narrow focus on resource shifts *within *a program budget. In either case, addressing the issues raised in the development of G-CEA might also be considered a pre-requisite for the use and re-use of the HsW model.

Several of these issues have been discussed above including: the availability of a suitable composite outcome measure that can support comparisons across disparate interventions and identifying political constraints on the set of possible resource shifts. However, the pivotal issue raised in development of the G-CEA model is the limited transferability of cost-effectiveness ratios in the presence of a complex series of dependencies between the costs and effects of related interventions. If cost-effectiveness is calculated relative to a comparator that is available in one population but not in another, then the resulting cost-effectiveness ratio will be applicable and policy-relevant in only one of those populations. In principle, evaluating sets of related interventions with respect to the 'null set' of related interventions (rather than with respect to the current mix of available interventions) would yield cost-effectiveness ratios that are independent of any such dependencies between related interventions.

After evaluating related interventions with respect to the null set of related interventions, the G-CEA model requires that independent sets of mutually exclusive interventions be ordered in increasing order of cost per unit of outcome, before applying standard decision rules for constrained maximisation. The G-CEA model can therefore be viewed as an adaptation of the QALY League Table approach, designed to enhance the transferability of results from one population to another. The G-CEA model de-contextualises CEA analyses, eschewing context-specific distributional concerns and taking only limited account of political constraints such that enhanced transferability may come at the price of policy-relevance. Decision-makers attempting to derive a context-specific set of priorities would then have to perform the usual adjustments to the price and quantity of inputs and to the coverage, efficacy and adherence of interventions [18], as well as conducting multiple indirect comparisons (when the null set is not politically feasible in the relevant context and to recover incremental comparisons against current practice) and re-weighting outcomes to reflect context-specific distributional concerns [22,23].

#### Program Budgeting and Marginal Analysis (PBMA)

is a relatively common approach to priority setting in the health sector, developed in the context of health agency decision making. The key features are; i) the establishment of a Working Group (from the health organisation and possibly other constituencies) to determine program objectives and to identify a set of services to be expanded and another set to be contracted, based on consideration of possible benefits (gained or lost) from service expansion or contraction, ii) estimation of budgets for sub-programs, and iii) calculation of cost-effectiveness ratios for each intervention in the expansion and contraction lists (optional).

The strength of this approach derives from the engagement of the key players in the task through the primary role of the Working Group, arguably increasing the likelihood that recommendations will be implemented. It is also possible to complete such an exercise at relatively low cost and with few data inputs. The key limitation of PBMA relates to the subjectivity of the process and consequent lack of confidence in the rankings and specifically in the expansion and contraction set identified. Peacock and colleagues [21], for example, report only a weak relationship between recommended Panel rankings and cost-effectiveness estimates prepared by the research team (even when measuring effectiveness to reflect objectives developed by the Panel). While it is possible that cost-effectiveness ratios do not adequately capture the range of social objectives that influencing rankings, it is also possible that rankings that rely heavily on subjective assessments will fail to reflect arguments in the social welfare function and will instead reflect decision-maker values. The latter is a particular concern given that the process has few requirements around the quality of the evidence-base and may be vulnerable to capture by vested interest.

Partly to address the limitations of a traditional PBMA analysis, Carter and colleagues have emphasised the importance of incorporating objective evidence into priority setting, whilst maintaining a core role for the Working Group. This work has progressed under the acronym of ACE: Assessment of Cost-Effectiveness (24, 25). Under ACE the incremental cost per DALY is adopted as the primary measure of performance, but 'second stage filters' such as 'acceptability', 'implementability', budget impact and equity are considered through a subjective process, possibly changing recommendations. The Working Group selects the intervention options and assists in the application of the 'second stage filters'. The research team prepares the cost/DALY estimates based on the clinical trial literature supported by economic and epidemiological modelling.

The ACE model has much in common with the HsW model, notably the disease focus and the greater reliance on objective evidence. Where it differs from the HsW model is in the role of the Working Group in selecting intervention options, rather than the use of objective criteria for this purpose, the adoption of the DALY as the primary measure of outcome, and the role for 'second stage filters' in the ranking of interventions to account for distributional concerns and to bring selected implementation issues into the priority setting exercise. While the ACE model does not allow the primary measure of outcome to be adjusted to account for non-health dimensions of benefit, the use of second-stage filters allows for other issues, including barriers to implementation that might modify the list of priorities to be made explicit. The selection of interventions by the Working Group remains as a source of inefficiency.

To date, the majority of PBMA exercises have set priorities at the level of relatively narrow program budgets that rarely include *all *mutually exclusive and complementary interventions for the relevant (sub) population [26]. Recently, the term macro marginal analysis (MMA) has been coined to describe the application of PBMA methods across disease-stages, disease-areas, modalities and program budgets administered by major integrated health care organisations [27]. The rationale for the development of MMA PBMA is much the same as for the development of the HsW model – to eliminate artificial constraints that entrench allocative inefficiency. However, the choice set is delimited firstly, by the ambit of the budget-holder (a constraint not present in the HsW model) and secondly, by the expansion and contraction lists compiled by the Working Group. It is worth noting that reluctance among program managers to nominate interventions for contraction has been identified as a key challenge in the implementation of MMA. Mitton and colleagues [27] argue that a recent implementation of MMA to identify desirable resource shifts for one regional health authority (Alberta, Canada) confirms that it is possible for a PBMA working group to pragmatically compare re-allocation options at the level of a regional health authority and achieve resource release of a significant magnitude without formal measurement of health benefits and without attaching explicit weights to competing objectives. While a comparison of re-allocation options without measurement and without a well-defined objective function is certainly possible, the more pertinent question would seem to be whether or not the resulting trade-offs are likely to leave people better-off or worse-off? The fact that resource shifts are acceptable to the Working Group provides little in the way of reassurance.

## Discussion

The primary strengths of the HsW model are: i) the health sector wide framework, ii) a means to stage the priority setting task to encompass all health interventions, that minimising the risk of sub-optimising, iii) the requirement for comprehensiveness in the range of interventions options – which reduces the influence of the status quo or vested interest on the scope of the priority setting exercise and ensures that priorities are set over all mutually exclusive and complementary interventions for the prevention or treatment of the disease/health problem under consideration; iv) the requirement for objective evidence; v) the separation of the technical priority setting task from implementation. The adoption of a disease focus has many advantages, including efficiency in the research activity, reduction in the number of target groups for interventions and a commonsense understanding of the recommendations.

Whilst the requirement for comprehensiveness in scope presents a specific challenge of the HsW model, our experience in applying the HsW model in a number of disease-areas has suggested a number of strategies in delimiting the set of interventions for formal performance measurement, whilst still meeting this requirement:

• Within a disease-area, interventions can be sorted into 'types' and evaluated as, for example, 'self-management interventions for type II diabetes' or 'brief GP interventions for problem drinking'. While it is true that there may be differences in the intensity and approach of specific interventions within each type, types can be defined so as to be homogeneous with respect to key characteristics such as program logic and this approach is more palatable than the PBMA approach to delimiting the choice set (ie, working group nominates expansion and contraction lists).

• Retaining all competing and complementary interventions of interest in the choice set but excluding interventions with insufficient evidence to support formal evaluation from the performance measurement task.

• Frequently 'simple modelling' will identify those interventions that can be considered highly cost-effective and those at the other end of the spectrum. Resources can then be reserved for detailed modelling of those interventions that fall somewhere in the middle.

Given such strategies, our experience suggests that application of the HsW model, within a single disease, will involve perhaps 25 cost-utility analyses and can be completed for around 5 person years of research: that is, for ~A$500,000 (or ~US$370,000). This would typically represent <0.05% of the resources allocated to management and prevention of the disease in question. Application of the HsW model typically results in clear recommendations for resource shifts between health services, across modalities and across disease stages. The highly disparate estimates of cost/QALY between interventions, means that the potential for health gain from recommended resource shifts is substantial. With opportunities identified to shift resources from interventions costing >$100,000/QALY gain to others costing <$10,000 per QALY gain, yielding potentially nine extra QALYs at no additional cost. Desirable resource shifts identified from application of the HsW model to osteoarthritis, Type 2 Diabetes, hypertension and harmful life style behaviours are specified elsewhere [9,11,12].

The loss in social benefit if recommendations are not followed can be calculated, as can the implied value (or cost) of non-health objectives and of political constraints. Keeping the priority setting exercise separate from issues of implementation is particularly important in this respect, supporting the development of clear recommendations based on the health care objectives identified in the priority setting exercise rather than political considerations. Priority setting models which attempt to identify and implement desirable resource shifts tasks in the one exercise; whilst possibly increasing the likelihood of implementation in the short term, may lose clarity with regards to objectives and decision criteria.

Key challenges in application remain. These challenges are not unique to the HsW model, but intrinsic to any attempt to compare performance across disparate interventions. Of the available alternative models, an evidence-based PBMA of the sort described in the ACE studies carries many of the advantages of the HsW model. However, one of the strengths of PBMA – its responsiveness to local objectives and constraints – may also leave the process open to capture by vested interest. That said, lessons learnt from the application and refinement of PBMA and the HsW model suggest that a comprehensive approach to priority setting across the care continuum and across modalities is likely to provide a shorter route to optimal allocation of health resources than the sort of revision at the margin or narrow program focus that characterises common approaches to priority setting.

## Competing interests

The author(s) declare that they have no competing interests.

## Authors' contributions

Segal prepared the original draft, which was based largely on her research program. Mortimer contributed through substantial edits and revisions. Both authors read and approved the final manuscript.
